# Supplementary figures and images for: The Effect of Xanthohumol Derivatives on Apoptosis Induction in Canine Lymphoma and Leukemia Cell Lines
Source: Int J Mol Sci. 2023 Jul 21;24(14):11724. doi: 10.3390/ijms241411724 (PMC10380916; doi:10.3390/ijms241411724)

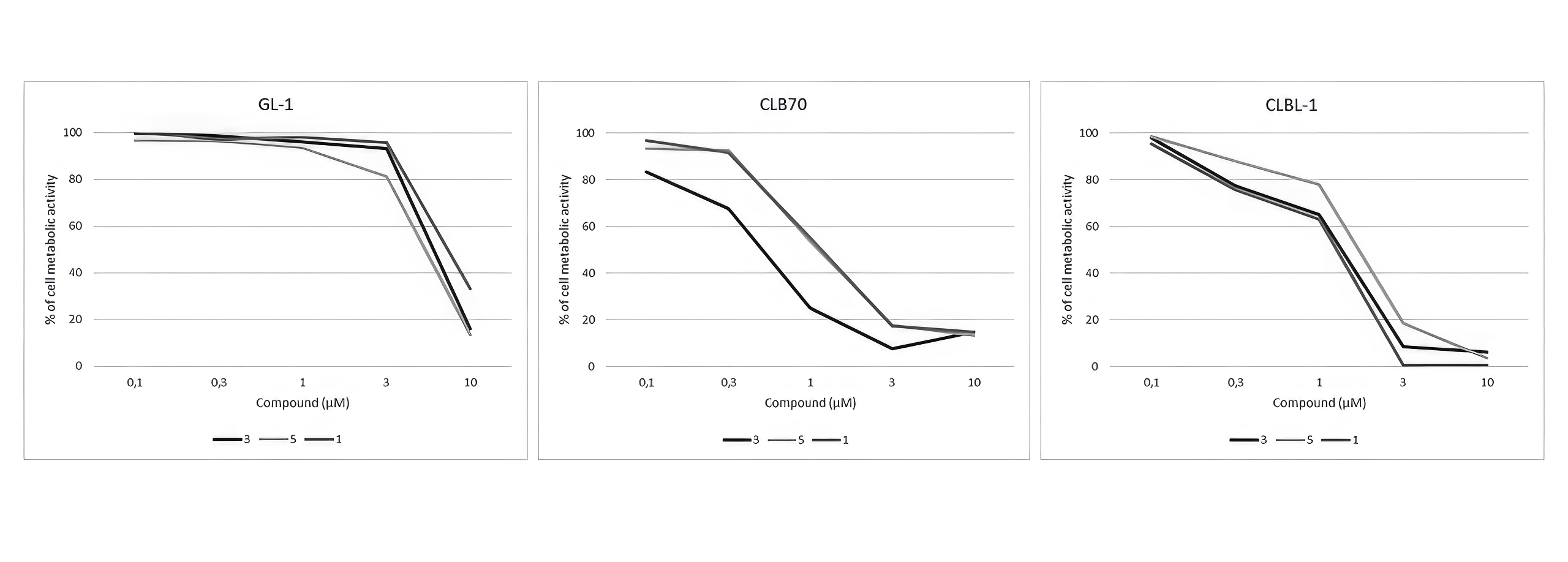

Supplement: Supplementary file 1 [file ijms-24-11724-s001.zip › ijms-2501389-supplementary.tif]
